# Long-term cardiometabolic effects of early institutionalization and foster care: Evidence from the Bucharest Early Intervention Project

**DOI:** 10.1017/S0954579426101540

**Published:** 2026-05-15

**Authors:** Michelle M.J. Mens, Ellen Jopling, Megan Hare, Stacy S. Drury, Charles A. Nelson, Charles H. Zeanah, Nathan A. Fox, Natalie Slopen

**Affiliations:** 1 Department of Social and Behavioral Sciences, Harvard T H Chan School of Public Health, Boston, MA, USA; 2 Boston Children’s Hospital/Harvard Medical School, Boston, MA, USA; 3 Department of Psychiatry and Behavioral Sciences, Tulane University, New Orleans, LA, USA; 4 Harvard Graduate School of Education, Harvard University, Cambridge, MA, USA; 5 Department of Human Development and Quantitative Methodology, University of Maryland, College Park, MD, USA; 6 Neuroscience and Cognitive Science Program, University of Maryland, College Park, College Park, MD, USA

**Keywords:** cardiometabolic health, catch up, early deprivation, foster care, longitudinal

## Abstract

Early psychological deprivation is associated with increased risk for cardiometabolic disorders, and rapid catch-up growth following improved care environments may further influence this risk. Using data from the Bucharest Early Intervention Project – a randomized longitudinal study of children institutionalized in infancy assigned to high-quality foster care, continued institutional care, or raised by biological families – we examined cardiometabolic outcomes in early adulthood. Participants were 81 women and 58 men (mean age 22.6 years), predominantly Romanian. We assessed associations between care type and cardiometabolic traits including blood pressure, lipid and glucose metabolism, and metabolic syndrome. Results indicated that adults with histories of institutionalization displayed poorer lipid metabolism compared to non-institutionalized controls. Notably, individuals randomized to foster care showed increased BMI and waist circumference in adulthood relative to those who remained in institutional care. Longitudinal latent class analyses revealed four distinct BMI trajectories from infancy to early adulthood. The accelerated BMI trajectory, predominantly comprising foster care participants, was associated with the greatest prevalence of metabolic syndrome. These findings suggest that both lipid dysregulation and persistent increases in BMI represent ongoing concerns for cardiometabolic risk following early psychosocial deprivation. Those exposed to early adverse care may benefit from ongoing cardiometabolic monitoring and lifestyle interventions.

Millions of children worldwide grow up in institutional care environments, such as orphanages, which often lack essential protective factors for healthy development, including individual attention, emotional support, and stable relationships. Psychosocial deprivation in institutional care is comparable to a severe form of neglect, the most common form of child maltreatment worldwide, which is estimated to affect 7.7 per 1,000 children per year in the United States (U.S. Department of Health & Human Services, 2023). Institutionalization and neglect can result in long-term negative effects, including impaired physical growth, delayed cognitive development, and increased risk for psychological disorders. (Bos et al., 2011; Golm et al., 2020; van Ijzendoorn et al., 2020). Transitioning from institutional care to a family-based setting, such as foster care, is associated with improved health outcomes in the years immediately following the move out of institutional environments (van Ijzendoorn et al., 2020). However, some affected children remain at risk for ongoing developmental challenges and chronic health conditions into adulthood (Sonuga-Barke et al., 2022). For example, adopted children who were exposed to institutionalized care often exhibit rapid catch-up growth, which is associated with an increased risk for cardiometabolic diseases (Tang et al., 2018). The Bucharest Early Intervention Project (BEIP), the only randomized controlled trial (RCT) of foster care as an alternative to institutional care, has followed participants from infancy into young adulthood. While insights from the BEIP indicate that institutionalization and foster care have a profound impact on various adverse outcomes in young adulthood, including executive functioning (Wade et al., 2023) and cognitive ability (Humphreys et al., 2022; Tan et al., 2023), the effect on cardiometabolic health remains unclear.

The prevalence of cardiometabolic diseases, including type 2 diabetes, is increasing globally, especially among adolescents and young adults (IDF Diabetes Atlas, 2025; Tönnies et al., 2023; Wagenknecht et al., 2023). Notably, earlier age at diabetes diagnosis is associated with a linear increase in mortality risk, including from cardiovascular disease (Emerging Risk Factors, 2023). Individuals who develop type 2 diabetes at a younger age are often characterized by higher body mass index (BMI), high blood pressure, dyslipidemia, and abnormal glucose function (Steinarsson et al., 2018; Wright et al., 2020). As these cardiometabolic risk factors are largely preventable, early detection, targeted interventions, and lifestyle guidance are critical to reducing long-term disease burden. In addition, previous research has found a robust negative association between adverse childhood events, such as childhood maltreatment, and cardiometabolic health (Chandan et al., 2020; Danese & Tan, 2014; Elsenburg et al., 2017; Rich-Edwards et al., 2010), suggesting that individuals with a history of early deprivation are at a higher risk for adverse cardiometabolic outcomes. For example, a recent study found that both emotional and physical neglect were strongly associated with high lipid levels in young adults (Kisely et al., 2023). Yet, most studies are based on retrospective cohorts where recall and response bias may occur. Prospective studies on institutionalization, like the BEIP, that follow participants over long periods allow us to assess developmental and long-term outcomes with a higher reliability and validity than retrospective studies. In an earlier study with the BEIP cohort, we investigated the effects of institutionalization and foster care interventions on cardiometabolic outcomes at age 16. While the latter results were null, a plausible possibility is that age 16 is too early to identify differences in cardiometabolic markers (Slopen et al., 2019).

## The current study

The current study had three aims. First, we estimated the association between institutionalization, intent to treat (randomization into foster care), and cardiometabolic traits – such as metabolic syndrome, blood pressure, lipid and blood-based markers, and anthropometric traits – among BEIP participants at age 22. Second, we examined variance in BMI and blood pressure changes between individuals and groups over the course of the BEIP, as captured in a longitudinal model from infancy to young adulthood. Third, we explored whether heterogeneous trajectories were present among the participants and whether they differ based on sex, ethnicity, and other cardiometabolic characteristics. Based on previous BEIP studies, we hypothesized that individuals who experienced institutional care in early life would display elevated cardiometabolic risk, as compared to participants who did not experience institutional care. Furthermore, previous BEIP studies focused on physical health trajectories from infancy to adolescence found that participants randomized to the foster care group (FCG) experienced greater increases in BMI (Johnson et al., 2018; Tang et al., 2018), with distinct trajectories correlated with higher levels of glycosylated hemoglobin (HbA1c) (Tang et al., 2018). Now that participants have reached adulthood, we hypothesized that distinct trajectories in BMI and blood pressure would emerge, influenced by early institutionalization and foster care placement, and that these trajectories would correlate with other cardiometabolic markers. Despite earlier increases in BMI, we further hypothesized that participants placed in foster care early in life would show overall lower cardiometabolic risk compared to those who remained in institutional care, reflecting the long-term benefits of early family-based intervention.

## Method

### Participants

The BEIP is a longitudinal RCT involving children raised in institutions from early infancy. The study recruited children from institutions for young children in Bucharest, Romania. Out of 187 institutionalized children screened for eligibility, 51 children were excluded due to medical reasons (e.g., genetic and fetal alcohol syndromes). Consequently, 136 children aged 6 to 30 months were included in the institutional care sample.

**Figure 1.**
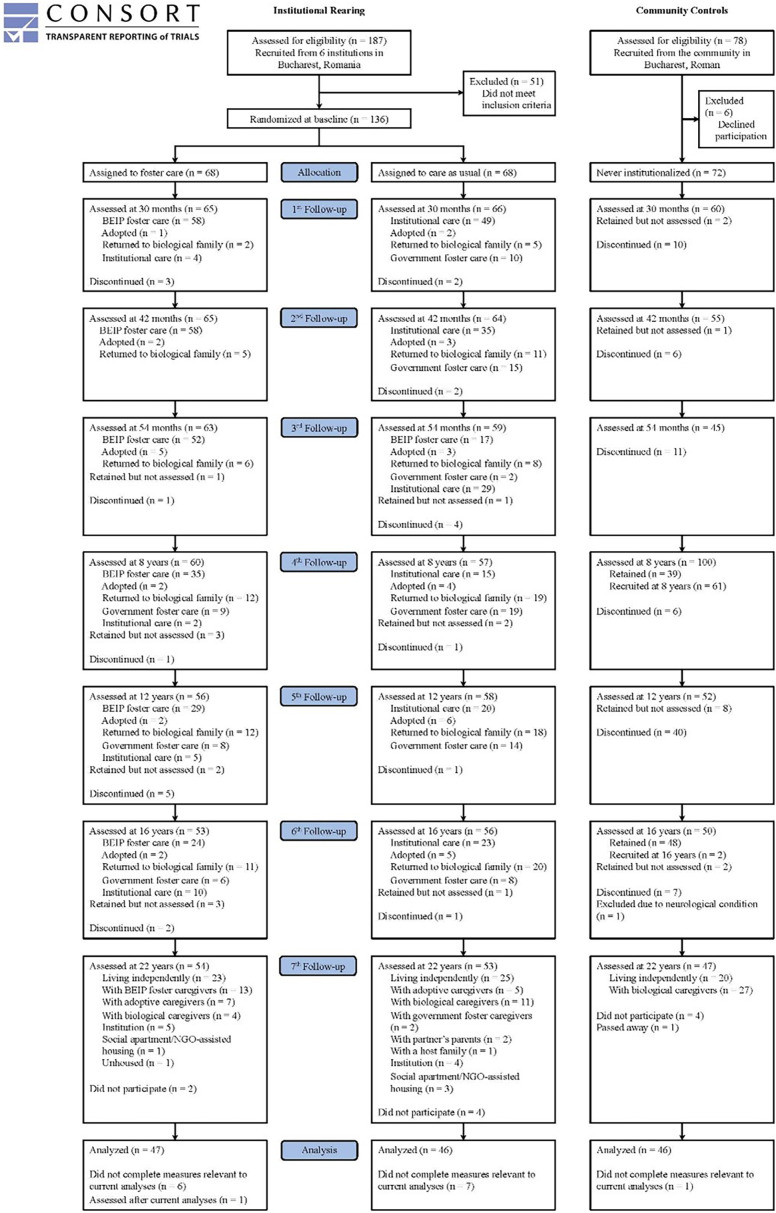
Flow chart of BEIP study participants.

At baseline, half of the children in institutions were randomly assigned to high-quality foster care developed and monitored by the BEIP team (FCG; *n* = 68), while the remaining children continued to receive standard institutional care (care as usual group [CAUG]; *n* = 68). The randomization process and intervention details have been described elsewhere (Zeanah et al., 2003). Foster parents were paid a salary in accordance with Romanian law and received support from BEIP’s team of social workers. Additionally, a sample of age- and sex-matched typically developing children was recruited from pediatric clinics (never-institutionalized group [NIG]; *n* = 72), with more children from the community included in subsequent follow-ups (Figure 1). At baseline, no significant differences were observed between the FCG and CAUG groups in terms of sex, birth weight, age, or the percentage of life spent in an institution.

The BEIP was initiated in collaboration with the Institute of Maternal and Child Health of the Romanian Ministry of Health, with rigorous procedures in place to ensure ethical integrity (Miller, 2009; Millum & Emanuel, 2007; Zeanah et al., 2006). The study protocol received approval from an ethics committee composed of appointees from Bucharest University and various government departments, as well as from local child protection commissions in Bucharest. Additionally, institutional review boards at the three home institutions of the primary investigators (C.H.Z., C.A.N., and N.A.F.) also approved the protocol. Informed consent was obtained from the children’s legal guardians, and assent was obtained from the children at ages 8, 12, and 16 for each procedure. At age 22, written or oral informed consent was obtained directly from the participants.

At age 22, a subgroup of the BEIP participants (*n* = 139: CAUG, *n* = 46; FCG, *n* = 47; NIG, *n* = 46) with available cardiometabolic data were included in the current study. There were no differences in sex (*p* = .97), ethnicity (*p* = .99), birth weight (*p* = .75), baseline age (*p* = .87), and baseline BMI (*p* = .99) between those with a history of institutionalization who provided cardiometabolic data and those who did not.

## Measures

### Cardiometabolic measurements

We assessed a variety of standard metrics of cardiometabolic health. Height and weight, measured while the participants were standing without shoes and heavy outerwear, were collected by a trained research assistant at all ages from baseline till age 22. BMI was calculated as weight divided by height squared (kg/m^2^). Age- and sex-standardized BMI *z*-scores were calculated according to the World Health Organization reference values. Waist circumference was measured at the level midway between the lower rib margin and the iliac crest with participants in standing position at age 22. Skinfold thickness was measured in triplet to the nearest 0.1 mm, using a skinfold caliper at three sides: biceps, triceps, and subscapular, at age 22. Mean values were obtained from the three measurements and total skinfold was calculated (Mbicep + Mtricep + Msubscapular). Systolic (SBP) and diastolic blood pressure (DBP) in mm Hg, were obtained in triplet by an automated monitor (Omron 7 Series) following a standard protocol where the participants are seated with feet on the floor; the average of the second and third readings were calculated and used in analyses at ages 12, 16, and 22. Pulse pressure (PP) was measured as the difference between SBP and DBP (mm Hg).

Blood was drawn after an overnight fast. A range of metabolic risk factors were measured at age 22. For lipids, the following parameters were assessed in mg/dL: total cholesterol (TC), high-density lipoprotein (HDL) cholesterol, and triglycerides (TGs). Low-density lipoprotein (LDL) was calculated based on the Friedewald equation (TC-HDL-TG/5) (Friedewald et al., 1972). HbA1c was obtained as a percentage of total hemoglobin and represents the average blood glucose levels over the past 2–3 months. Fasting blood glucose was measured in mg/dL. Measurement of fasting blood glucose was excluded and imputed using multiple imputation when non-compliance with fasting was indicated (*n* = 1).

Metabolic syndrome score was calculated follow standard practices, with participants receiving one point for each value above the following thresholds: a waist circumference of more than 94 cm in men and 80 cm in women; serum TGs levels of 150 mg/dL or greater; HDL levels less than 40 mg/dL in men or less than 50 mg/dL in women; fasting glucose levels of 100 mg/dL or higher; and SBP of 130 mm Hg or higher and/or DBP of 85 mm Hg or higher (Grundy et al., 2005). Scores ranged between 0 and 5. Diagnosis of metabolic syndrome was defined as having exceeded the risk threshold for at least three out of the five criteria (Grundy et al., 2005). We calculated continuous metabolic syndrome scores by summing standardized *z*-scores of each measure, with HDL reverse coded to reflect higher risk.

### Covariates

Information on sex, smoking status (at least one cigarette/e-cigarette every day for 30 days), ethnicity (Roma vs. non-Roma), and medication use (i.e., blood pressure lowering medication, blood glucose lowering medication) were obtained through questionnaires.

### Statistical analysis

Sample characteristics are presented as mean ± SD or median ± IQR for continuous variables, and categorical variables are expressed as numbers and percentages. All statistical analyses were performed in R.4.3.1. Missing data was imputed using multiple imputation (12.2% incomplete cases) using the R package “mice” (*n* = 10 iterations).

### Cardiometabolic risk factors and metabolic syndrome

We compared unadjusted medians and means for each cardiometabolic risk factor (i.e., BMI-*z*, waist circumference, skinfold thickness, SBP, DBP, PP, TC, HDL-cholesterol, TG, LDL-cholesterol, Hb1Ac, and fasting glucose), and for composite metabolic risk (i.e., metabolic syndrome, and *z*-values of metabolic score), to estimate group differences between children with a history of institutionalization (EIG) and never institutionalization (NIG), and between children randomized to the FCG and CAUG. The unadjusted group comparisons were analyzed using a Student *t* test for means, or a Wilcoxon rank sum test for medians. In adjusted models, linear regression was used for continuous outcomes adjusted for sex, smoking status, ethnicity, and medication use. For binary outcomes (e.g., metabolic syndrome), logistic regression was used.

### Variable-centered trajectories

Variable-centered trajectories of BMI *z*-scores and blood pressure *z*-scores (SBP, DBP, PP) were analyzed using linear mixed-effects models within a multilevel framework in R (“lme4” package). The dependent variables included repeated measurements of BMI *z*-scores (i.e., baseline to 22 years) and blood pressure *z*-scores (i.e., 12, 16, and 22 years). The inclusion criteria for this repeated measurement analysis required individuals to have data on the 22-year assessment and data on at least two timepoints over the course of the study. Accordingly, the analysis on BMI trajectories included 132 individuals (CAUG, *n* = 46; FCG, *n* = 46; NIG, *n* = 40) with a total of 813 datapoints, and the analysis on the blood pressure trajectories included 118 individuals (CAUG, *n* = 41; FCG, *n* = 44; NIG, *n* = 33) with a total of 310 datapoints.

First, unconditional models with a random intercept and fixed slope were tested using polynomial functions of age (age, age^2^, and age^3^) across the entire sample to determine the best-fitting growth function for BMI, SBP, DBP, and PP. Model fit indices (e.g., log-likelihood, Akaike information criterion [AIC], Bayesian information criterion [BIC]) were highly similar across all three models, indicating that more complex models did not significantly improve fit compared to a linear model. Therefore, a linear growth model was selected for all four outcome variables (fit indices provided in Table S1).

Next, the analysis proceeded in four steps using the linear model. First, an intercept-only model was estimated to assess the proportion of between- and within-person variance in each outcome. Second, an unconditional growth model was specified, including age at assessment (person-mean centered) as a predictor with both random intercept and random slopes. Third, group effects were added as fixed predictors. Finally, sex and Roma status were included as additional time-invariant covariates.

To compare differences in intercepts and slopes, separate models were conducted to estimate contrasts between EIG and NIG (reference) and between FCG and CAUG (reference) in the final model.

### Person-centered trajectories

Latent class growth analysis (LCGA) was conducted using the “lcmm” package in R. A linear function was specified to identify heterogeneous trajectories of person-mean centered BMI *z*-scores across time (from baseline to 22 years), and SBP, DBP, and PP *z*-scores (measured at ages 12, 16, and 22 years). Age was centered at the first timepoint. LCGA facilitates the classification of individuals according to their most likely trajectory, although findings should be interpreted with caution, as larger sample sizes are generally preferable when using this technique. (Sinha et al., 2021). Models with two to five classes were evaluated using full information maximum likelihood estimation (FIML; *n* = 50 iterations), which allows for inclusion of participants with missing data and is designed to provide unbiased estimates. To determine which model best captured the underlying structure of our data, we considered several statistical fit indices, including AIC, BIC, and sample size-adjusted BIC (ssaBIC). Lower values on these indices are indicative of better model fit, with AIC generally favoring simpler models and BIC/ssaBIC imposing greater penalties for additional parameters. Model classification accuracy was assessed using entropy; values closer to 1.0 indicate more distinct separation between classes, with values above 0.8 typically considered acceptable. Given our modest sample sizes (*n* = 132 for BMI and *n* = 118 for blood pressure), we required that each identified trajectory group comprise at least 5% of participants to ensure adequate class stability and interpretability. Finally, after the optimal number of classes was determined, we cautiously explored associations between the identified subgroups and other assessed cardiometabolic risk indicators.

### Post hoc sensitivity analysis time of placement

Within the FCG, we conducted secondary analyses to test whether age at foster care placement was associated with BMI trajectories. Individuals were divided into “younger-placement” and “older-placement” subgroups based on a median split on age at foster care entry (775 days; 2.12 years). We then fit a linear mixed-effects model of BMI *z*-scores including placement subgroup, timepoint (centered at the first assessment, time = 0), and their interaction, with gender and ethnicity as covariates, and random intercepts and slopes to account for within-child variability over time.

## Results

Cohort characteristics of study participants for the 22-year assessment are described in Table 1. Neither age, sex, smoking nor medication use differed across groups. The EIG group included a higher proportion of Roma participants compared to the NIG (*p* = .001). Besides a moderate negative correlation between sex and pulse pressure (*r* = −.62), other cardiometabolic traits were not correlated with sex (Figure S1).

**Table 1.** Characteristics of study participants at 22-year assessment

Group	Ever institutionalized	Never institutionalized	*p*-Value	Foster care intervention	Care as usual	*p*-Value
*n*	93	46		47	46	–
Age (mean (SD))	22.60 (1.09)	22.70 (1.17)	0.626	22.52 (1.01)	22.67 (1.18)	0.512
Ethnicity, *n* (%)	–	–	0.001	–	–	0.345
*Romanian*	59 (63.4)	44 (95.7)	–	33 (70.2)	26 (56.5)	–
*Roma*	32 (34.4)	2 (4.3)	–	14 (29.8)	18 (39.1)	–
* Hungarian*	1 (1.1)	0 (0.0)	–	0 (0.0)	1 (2.2)	–
* Other*	1 (1.1)	0 (0.0)	–	0 (0.0)	1 (2.2)	–
Female, *n* (%)	49 (52.7)	32 (69.6)	0.086	25 (53.2)	24 (52.2)	1.000
Smoking, *n* (%)	59 (63.4)	34 (73.9)	0.297	29 (61.7)	30 (65.2)	0.891
Medication, *n* (%)	6 (6.5)	2 (4.3)	0.909	2 (4.3)	4 (8.7)	0.653
BMI (mean (SD))	25.13 (6.03)	25.11 (5.11)	0.984	26.50 (6.82)	23.73 (4.79)	**0.026**
Waist circumference (mean (SD))	85.80 (14.50)	82.44 (12.73)	0.183	89.09 (16.23)	82.43 (11.74)	**0.026**
Skinfold thickness (mean (SD))	43.51 (25.25)	46.62 (21.66)	0.477	47.65 (27.80)	39.28 (21.85)	0.11
Systolic blood pressure (mean (SD))	111.53 (12.99)	108.09 (11.04)	0.125	111.69 (12.37)	111.37 (13.73)	0.906
Diastolic blood pressure (mean (SD))	71.79 (9.53)	73.62 (10.17)	0.300	73.41 (10.40)	70.13 (8.34)	0.097
Pulse pressure (mean (SD))	39.74 (12.23)	34.47 (10.92)	**0.014**	38.28 (12.78)	41.24 (11.58)	0.245
Total cholesterol (mean (SD))	163.59 (36.38)	170.21 (27.42)	0.278	169.01 (37.89)	158.06 (34.28)	0.148
HDL-cholesterol (mean (SD))	46.37 (11.58)	54.29 (12.58)	**<0.001**	46.16 (11.35)	46.60 (11.93)	0.856
LDL-cholesterol (mean (SD))	97.79 (31.90)	98.07 (25.49)	0.958	102.64 (33.84)	92.83 (29.33)	0.139
Triglycerides (mean (SD))	97.18 (50.37)	89.23 (52.75)	0.390	101.10 (47.94)	93.17 (52.97)	0.451
Hb1Ac (mean (SD))	5.38 (0.24)	5.37 (0.41)	0.856	5.41 (0.24)	5.35 (0.24)	0.247
Fasting glucose (mean (SD))	73.48 (9.68)	74.74 (8.76)	0.458	74.49 (10.68)	72.45 (8.54)	0.313
Metabolic syndrome, *n* (%)	12 (12.9)	5 (10.9)	0.945	9 (19.1)	3 (6.5)	0.132
Metabolic z-score (mean (SD))	0.04 (0.57)	−0.07 (0.59)	0.308	0.14 (0.63)	−0.08 (0.48)	0.062

Significant *p* values are highlighted in bold.

BMI = body mass index, HDL-cholesterol = high-density lipoprotein cholesterol, LDL-cholesterol = low-density lipoprotein cholesterol, Hb1Ac = glycosylated hemoglobin.

## Association between institutionalization, intent to treat, and cardiometabolic traits

### Institutional rearing effects

In unadjusted models, individuals with a history of institutionalization (EIG group) demonstrated, on average, lower HDL-cholesterol levels (*p* < .01) and higher PP (*p* = .01) compared to those without such a history (NIG group). After adjusting for covariates, HDL-cholesterol levels remained significantly lower in the EIG group (*p* = .01), whereas the difference in PP was no longer significant. Additionally, the adjusted model revealed significantly lower TC levels in the EIG group compared to the NIG group (*p* = .03). Other cardiometabolic traits did not differ significantly between the EIG and NIG groups (Table 2).

**Table 2.** Effects of institutionalization and foster care intervention on cardiometabolic traits

	Effect of history of institutionalization on cardiometabolic traits						Effect of foster care intervention on cardiometabolic traits					
	*Unadjusted*			*Adjusted*			*Unadjusted*			*Adjusted*		
Outcome^†^	Beta	Std error	*p* Value	Beta	Std error	*p* Value	Beta	Std Error	*p* Value	Beta	Std Error	*p* Value
BMI	−0.02	1.04	.98	0.11	1.13	.92	2.76	1.22	**.03**	2.77	1.26	**.03**
Waist circumference	−3.36	2.51	.18	−1.04	2.64	.69	6.66	2.94	**.03**	6.93	2.94	**.02**
Skinfold thickness	3.11	4.35	.48	2.19	4.58	.63	8.37	5.19	.11	8.42	5.04	.10
Systolic blood pressure	−3.45	2.23	.13	−1.02	2.13	.63	0.32	2.71	.91	0.70	2.40	.77
Diastolic blood pressure	1.83	1.76	.30	1.33	1.90	.49	3.28	1.96	.10	3.14	2.00	.12
Pulse pressure	−5.27	2.13	**.01**	−2.35	1.85	.21	−2.96	2.53	.24	−2.44	2.04	.23
Total cholesterol	6.62	6.07	.28	14.03	6.30	**.03**	10.95	7.50	.15	13.45	7.18	.06
HDL-cholesterol	7.92	2.15	**.00**	6.31	2.24	**.01**	−0.44	2.41	.86	−0.50	2.39	.84
LDL-cholesterol	0.29	5.40	.96	8.01	5.61	.16	9.80	6.57	.14	12.56	6.26	.05
Triglycerides	−7.95	9.22	.39	−1.49	9.46	.88	7.93	10.47	.45	6.89	10.11	.50
Hb1Ac	−0.01	0.06	.86	0.03	0.06	.63	0.06	0.05	.25	0.06	0.05	.23
Fasting glucose	1.26	1.69	.46	−0.22	1.81	.90	2.04	2.01	.31	1.51	2.01	.45
Metabolic syndrome	−0.02	0.06	.73	−0.01	0.06	.89	0.13	0.07	.07	0.12	0.07	.08
Metabolic *z*-score	−0.11	0.10	.31	−0.11	0.11	.32	0.22	0.12	.06	0.21	0.12	.08

Significant *p* values are highlighted in bold.

*Models are adjusted for gender, ethnicity (Roma), smoking, and any medication use.

BMI; body mass index, HDL-cholesterol; high-density lipoprotein cholesterol, LDL-cholesterol; low-density lipoprotein cholesterol, Hb1Ac; glycosylated hemoglobin.

† Cardiometabolic outcome measures are from 22-year assessment.

### Intent to treat effects

When comparing individuals randomized to foster care (FCG group) with those who remained in institutional care (CAUG group), the unadjusted models showed that individuals in the FCG had, on average, a higher BMI (*p* = .03) and waist circumference (*p* = .03) than those in the CAUG group. Although not statistically significant (*p* = .06), a marginally higher prevalence of metabolic syndrome was observed in the FCG compared to the CAUG group. After adjusting for covariates, both BMI (*p* = .03) and waist circumference (*p* = .02) remained significantly higher in the FCG group, and there was a suggestive difference in metabolic syndrome, with the FCG group showing a higher prevalence (*p* = .08). Additionally, LDL-cholesterol levels were higher (*p* = .05) in the FCG group compared to the CAUG group after covariate adjustment (Table 2).

### Timing effects

Among the FCG participants, an older age at foster care placement was associated with higher Hb1Ac (*p* = .02), but not with the other cardiometabolic traits (Table S2). Duration of time in institutional care was not associated with cardiometabolic traits (Table S2). In a post hoc analysis among FCG participants, the younger- and older-placement subgroups did not differ in BMI *z*-score at the first assessment (adjusted means: 0.05 and 0.02, respectively; both confidence intervals included 0). Over time, however, their trajectories diverged. The younger-placement subgroup exhibited a modest increase in BMI *z*-scores (slope = 0.03 SD per timepoint, 95% CI = −0.00 to 0.05), whereas the older-placement subgroup showed a flat to slightly decreasing pattern (slope = −0.01 SD per timepoint, 95% CI = −0.04 to 0.02). Although the placement subgroup × time interaction was in the hypothesized direction, it did not meet conventional thresholds for statistical significance (*p* = .11).

### Cardiometabolic trajectories by group

**Figure 2.**
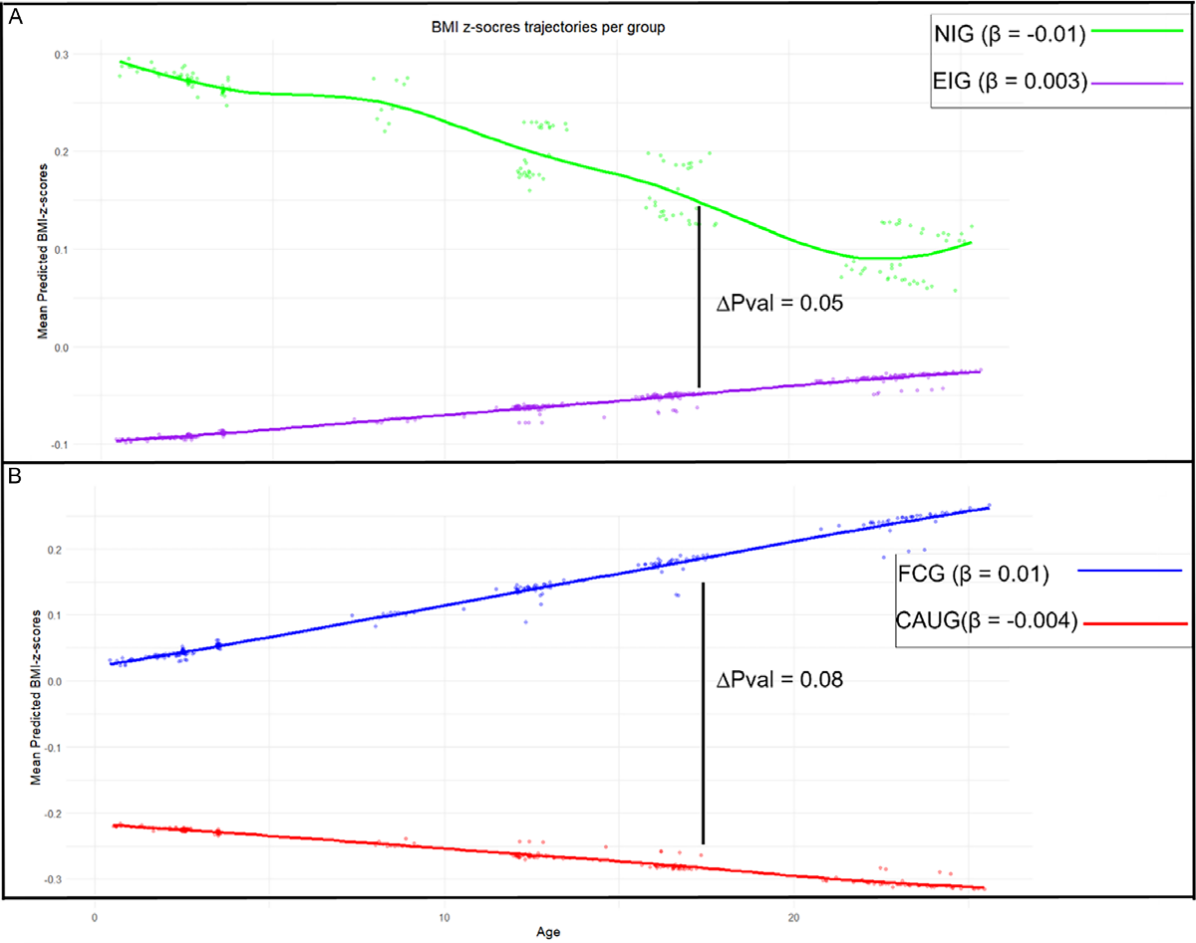
Mean predicted BMI *z*-scores over the course of the study stratified by group. The *β* illustrates the slope of the corresponding trajectory. The significance level of the slope comparison is illustrated by the ΔP value. Panel A presents the group comparison between never-institutionalized group (NIG) and ever institutionalized group (EIG). Panel B presents the group comparison between foster care group (FCG) and care as usual group (CAUG).

Figure 2 illustrates BMI *z*-score trajectories throughout the study for each group, adjusted for gender and Roma. At baseline, the EIG had a lower BMI compared to the NIG (*p* = .05) and showed a slight increase in BMI over time (*β* = 0.003), whereas the NIG showed a modest decline (*β* = −0.01). However, the rate of change did not significantly differ between groups (*p* = .09) (Figure 2). Within the EIG, the FCG had a higher baseline BMI than the CAUG (*p* = .02) and demonstrated an increasing BMI trajectory over time (*β* = 0.01), while the CAUG experienced a slight decline (*β* = −0.004); yet the difference in rates of change was not statistically significant (*p* = .08) (Figure 2B). Results of the LMM can be found in Table S3.

Regarding the blood pressure measures, no difference was found for all blood pressure measures in either the intercept as the rate of change between EIG and NIG, nor between the FCG and CAUG (Table S4, Table S5, Table S6).

### Person-centered BMI trajectories

**Figure 3.**
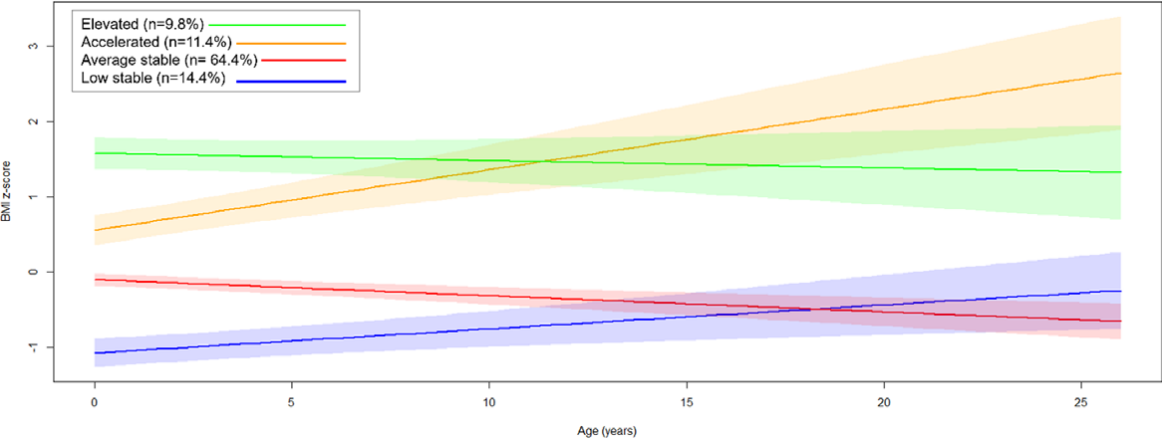
Four identified trajectories of the estimated person-mean BMI changes from baseline to age 22. Latent growth models identified four heterogeneous trajectories of person-mean centered BMI changes over the course of the BEIP study. The largest group is illustrated by the average stable (*n* = 85, 64.4%), followed by the low-stable group (*n* = 19, 14.4%), the accelerated group (*n* = 15, 11.4%), and the elevated group (*n* = 13, 9.8%).

Fit indices suggested that a four-class model provided the best fit, identifying heterogeneous BMI trajectories (Figure 3, Table S7): low-stable (*n* = 19, 14.4%), average-stable (*n* = 85, 64.4%), accelerated (*n* = 15, 11.4%), and elevated (*n* = 13, 9.8%). Most individuals in the accelerated trajectory are FCG (60%, *n* = 9), and 46.2% of those in the elevated trajectory are also FCG (*n* = 6); yet there is no statistically significant difference in group distribution across identified classes (*p* = .14). Although not reaching significance, there was a trend toward differences in female representation across the trajectory classes (*p* = .051), with the low-stable class showing the highest proportion of females (78.9%) and the average-stable class the lowest (48.2%).

BMI trajectory groups significantly differed in waist circumference (*p* < .001), skinfold thickness (*p* < .001), DBP (*p* = .01), PP (*p* = .004), HDL-cholesterol (*p* = .001), LDL-cholesterol (*p* = .02), TG (*p* = .001), metabolic syndrome (*p* = .005), and metabolic *z*-score (*p* < .001). The accelerated trajectory was characterized by the highest waist circumference, skinfold thickness, and metabolic syndrome prevalence, along with the lowest HDL-cholesterol levels. Meanwhile, the elevated trajectory had the highest DBP, TG levels, and metabolic score, and the lowest PP (Table 3).

**Table 3.** Characteristics of heterogeneous BMI trajectories

	Low stable	Average stable	Accelerated	Elevated	*p* Value
*n*	19	85	15	13	
Age (mean (SD))	22.51 (1.38)	22.70 (1.07)	22.42 (1.01)	22.21 (1.00)	.426
Group, *n* (%)					.136
* FCG*	5 (26.3)	26 (30.6)	9 (60.0)	6 (46.2)	
* CAUG*	10 (52.6)	31 (36.5)	3 (20.0)	2 (15.4)	
* NIG*	4 (21.1)	28 (32.9)	3 (20.0)	5 (38.5)	
Ethnicity, *n* (%)					.994
* Romanian*	15 (78.9)	61 (71.8)	10 (66.7)	10 (76.9)	
* Roma*	4 (21.1)	22 (25.9)	5 (33.3)	3 (23.1)	
* Hungarian*	0 (0.0)	1 (1.2)	0 (0.0)	0 (0.0)	
* Other*	0 (0.0)	1 (1.2)	0 (0.0)	0 (0.0)	
Female, *n* (%)	15 (78.9)	41 (48.2)	10 (66.7)	9 (69.2)	.051
Smoking, *n* (%)	9 (47.4)	59 (69.4)	9 (60.0)	10 (76.9)	.23
Medication, *n* (%)	3 (15.8)	4 (4.7)	0 (0.0)	0 (0.0)	.122
BMI (mean (SD))	16.80 (4.70)	18.24 (3.36)	22.63 (7.57)	24.60 (7.86)	<.001
Waist circumference (mean (SD))	75.11 (11.23)	81.98 (10.42)	103.92 (11.34)	98.67 (14.41)	<.001
Skinfold thickness (mean (SD))	35.21 (19.13)	38.79 (18.47)	72.74 (27.29)	65.95 (30.57)	<.001
Systolic blood pressure (mean (SD))	105.42 (10.70)	112.24 (13.05)	109.20 (12.67)	111.35 (9.05)	.176
Diastolic blood pressure (mean (SD))	70.89 (8.68)	71.03 (9.24)	75.77 (10.39)	79.58 (11.01)	.011
Pulse pressure (mean (SD))	34.53 (10.08)	41.21 (11.85)	33.43 (8.34)	31.77 (14.56)	0.004
Total cholesterol (mean (SD))	158.04 (26.02)	162.06 (32.41)	175.71 (34.84)	184.22 (42.49)	0.062
HDL-cholesterol (mean (SD))	56.97 (13.35)	48.86 (12.25)	40.14 (6.53)	45.12 (11.24)	0.001
LDL-cholesterol (mean (SD))	86.33 (25.36)	95.44 (29.47)	111.25 (30.73)	112.28 (31.01)	0.023
Triglycerides (mean (SD))	73.72 (27.41)	88.76 (48.08)	121.59 (51.85)	134.07 (69.42)	0.001
Hb1Ac (mean (SD))	5.46 (0.55)	5.35 (0.24)	5.39 (0.26)	5.48 (0.28)	0.365
Fasting glucose (mean (SD))	75.27 (9.65)	74.42 (8.85)	68.77 (9.06)	75.94 (12.11)	0.128
Metabolic syndrome, *n* (%)	0 (0.0)	8 (9.4)	5 (33.3)	4 (30.8)	0.005
Metabolic *z*-score (mean (SD))	−0.29 (0.52)	−0.09 (0.50)	0.44 (0.54)	0.60 (0.56)	<0.001

Significant *p* values are highlighted in bold.

BMI = body mass index, HDL-cholesterol = high-density lipoprotein cholesterol, LDL-cholesterol = low-density lipoprotein cholesterol, Hb1Ac = glycosylated hemoglobinCardiometabolic outcome measures are from 22-year assessment.

### Person-centered blood pressure trajectories

For SBP, model fit indices indicated that a three-class solution provided the best fit, capturing heterogeneity in intercept but not in slopes (Figure S2, Table S8). The largest class comprised individuals with an average intercept (*n* = 57, 48.3%), followed by those with the lowest intercept (*n* = 50, 42.4%), and a smaller class with the highest intercept (*n* = 11, 9.3%). There was no significant difference in sex (*p* = .54), or group distribution (CAUG, FCG, NIG, *p* = .89) across the three identified classes. Individuals in the highest intercept class had significantly higher DBP (*p* < .001), PP (*p* < .001), and metabolic *z*-scores (*p* < .001) compared to the other two classes (Table S9).

For DBP, a two-class model provided the best fit, again capturing differences in intercepts but not in slopes (Figure S3, Table S10). Most participants belonged to the class with a lower intercept (*n* = 97, 82.2%), while a smaller class had a relatively higher intercept (*n* = 22, 17.8%). Compared to the lower-intercept class, individuals in the higher-intercept class had significantly greater BMI (*p* < .001), waist circumference (*p* < .001), skinfold thickness (*p* = .009), SBP (*p* < .001), TGs (*p* = .005), metabolic syndrome (*p* = .001), and metabolic *z*-score (*p* < .001), along with lower HDL-cholesterol levels (*p* = .02) (Table S10). There were no significant differences in sex distribution across the identified classes (*p* = .24). Group distribution across the two classes did not differ significantly (*p* = .12), though FCG participants showed a nonsignificant trend toward overrepresentation in the class with a higher intercept (*n* = 12, 57.1%).

A two-class model also provided the best fit for PP trajectories, capturing differences in intercepts but not in slopes (Figure S4, Table S8). Consistent with the DBP trajectories, most participants fitted to the lower-intercept class (*n* = 92, 78.0%). The two classes differed significantly only in SBP, with the higher-intercept group exhibiting significantly higher SBP compared to the lower-intercept group (*p* < .001) (Table S11). There were no significant differences in sex distribution across the identified classes (*p* = .14). Group representation did not significantly differ across the two classes (*p* = .33), suggesting a comparable distribution of FCG, CAUG, and NIG participants between the two identified classes.

## Discussion

In a longitudinal cohort spanning two decades, we prospectively examined the relations between early psychological deprivation and randomization to foster care on cardiometabolic health outcomes in young adulthood. This study is the first to assess a comprehensive range of cardiometabolic markers within this cohort up to 22 years of age. Group difference indicated a disadvantage of lipid metabolism among those with a history of institutionalization, which may be driven by those who were randomized into foster care. Additionally, individuals assigned to foster care showed elevated BMI and waist circumference in early adulthood, with central adiposity levels exceeding clinical thresholds for increased cardiometabolic risk (waist circumference >94 cm in men and >80 cm in women). Longitudinal person-centered BMI trajectories revealed four distinct classes from infancy to young adulthood. The identified accelerated group, predominantly represented by FCG individuals, showed the highest prevalence of metabolic syndrome, signaling early vulnerability in this group to cardiometabolic risk and highlighting the need for targeted monitoring and intervention.

Our results build upon a prior BEIP study using data from age 16 that investigated metabolic and immune markers (Slopen et al., 2019), along with BMI trajectories (Tang et al., 2018). The longer follow-up and addition of measures of central adiposity, body fat, and lipid metabolism in the current study allowed for an extended and more detailed examination of cardiometabolic health and enabled us to define cardiometabolic disorder among participants (Grundy et al., 2005). Early adulthood is a critical window when long-term cardiometabolic dysfunctions may start to emerge, particularly among those with a history of childhood adversity (Bister et al., 2022; Danese et al., 2009; Kulak et al., 2024; Ruiz et al., 2018). In line with our results, prior research using a sample of young adults with histories of foster care and an age-matched sample drawn from the National Longitudinal Study of Adolescent to Adult Health demonstrated that young adults with a history of foster care exhibited significantly greater BMI at age 25 compared to non-exposed individuals (Ahrens et al., 2014).

Research from other cohorts suggests that not all cardiometabolic risks may emerge at the same time: while some may appear earlier, others may emerge later. For instance, a prospective study of children exposed to parental imprisonment showed elevated BMI beginning at age 14, but elevated SBP and DBP only emerged at age 30 (Roettger et al., 2022). Furthermore, another study found that cumulative adversity in the first seven years of life is associated with elevated BMI, but not blood pressure during adolescence (Slopen et al., 2014).

This aligns with our findings, given that we see early signs of dysregulated anthropometric traits, but no group differences in blood pressure, suggesting that age 22 may still be too early for blood pressure alterations to become detectable. Notably, most previous studies do not distinguish between different forms of childhood adversity. However, neglect, which is particularly prevalent in those with a history of institutional care, has been linked to distinct stress physiology, including dysregulation of the hypothalamic–pituitary–adrenal (HPA) axis (Humphreys et al., 2015; McLaughlin et al., 2014), especially in ways that could affect metabolic regulation.

Individuals randomized to foster care showed higher BMI and waist circumference when compared to those who remained in institutional care (CAUG). Furthermore, group-centered trajectories indicated an increase of BMI over the course of the study for the FCG. Taken together with our primary FCG vs. CAUG models, which showed that FCG participants overall had higher BMI than CAUG across time, the post hoc analyses of age at placement suggest that this increase in BMI is largely driven by children placed into foster care at a younger age. In contrast, those placed at an older age exhibit more stable and comparatively lower BMI trajectories, resembling those of the CAUG group. Although underpowered and only trend-level statistically, these findings are consistent with the possibility that age of foster care placement contributes to the observed group differences in BMI trajectories. This increase in BMI could potentially be interpreted as a form of delayed catch-up growth, a phenomenon often observed in children who experienced accelerated growth to recover to their expected growth trajectories after experiencing periods of stress or deprivation (Prader et al., 1963). In the context of foster care, the transition to a more nurturing environment with possibly better access to nutrition and care may contribute to this rapid growth. Prior BEIP studies have found evidence suggestive of catch-up growth. For instance, 24% of EIG participants were born with low birth weights (<2,500 g), compared to 3% among the NIG. In a follow-up study extending to 54 months, only those in the FCG showed substantial growth in both height and weight, and all FCG participants were within the normal range of growth trajectories 12 months after randomization (Johnson et al., 2010). Another BEIP study using follow-up into adolescence found that children assigned to foster care were overrepresented in an accelerated BMI trajectory from baseline to age 16, and this trajectory correlated with elevated levels of HbA1c and C-reactive protein at age 16. These findings suggest that the catch-up growth among FCG may lead to increased cardiometabolic and immune risk during adolescence (Tang et al., 2018). The fact that we see an overrepresentation of FCG in the accelerated BMI trajectory up to the age of 22 suggests that those individuals are consistently at risk for cardiometabolic health outcomes and should be monitored closely. In contrast to Tang et al’s 16-year follow-up, our accelerated BMI trajectory was not marked by elevated HbA1c, an indicator of average blood glucose over the past 2–3 months (Tang et al., 2018). The practical implication of this research is that pediatric healthcare providers and nutritionists should guide adoptive parents in supporting healthy catch-up growth by encouraging balanced nutrition, promoting regular physical activity, and educating them about the risks of rapid post-restriction weight gain – ideally starting these measures early in the child’s adjustment.

For the first time in BEIP assessments, waist circumference – a key indicator of visceral fat – and skinfold thickness – a measure of overall body fat – were collected. Levels were notably higher in the FCG group compared to CAUG, exceeding low-risk thresholds. Both the accelerated and elevated BMI trajectories were correlated with higher waist circumference and skinfold thickness. Waist circumference is a recommended clinical measure for cardiometabolic risk, predicting morbidity and mortality while complementing BMI for patient management (Ross et al., 2020). It is strongly linked to visceral fat accumulation around abdominal organs, disrupting normal endocrine functions more than subcutaneous fat (Dam et al., 2016; Després & Lemieux, 2006). This includes secretion of adipokines and pro-inflammatory cytokines, promoting inflammation and insulin resistance, key factors in metabolic syndrome, type 2 diabetes, and cardiovascular diseases. In this study, it is notable that the elevated BMI trajectory group displayed a healthy BMI, at the same time as showing high waist circumference. Literature shows that individuals with a healthy weight, but high visceral fat, are at higher cardiovascular risk than those with a higher BMI (Canoy et al., 2007; Stefan & Schulze, 2023). In future studies, it would be valuable to track visceral fat over time in relation to cardiometabolic disease outcomes, particularly among individuals with a history of institutionalized care. These findings should also be interpreted within the broader context of Romania’s post-revolution political transition, with consequences for changes in both economic and nutritional contexts. Obesity has risen substantially since the early 1990s, consistent with broad trends across Eastern Europe (Ulijaszek & Koziel, 2007), and current estimates suggest that approximately one quarter of children have overweight or obesity (Drăgănescu et al., 2025). The elevated levels of overweight and obesity observed in Romanian children may be attributed to several factors, including socioeconomic conditions and the types of foods and leisure-time physical activities that are available and preferred (Mocanu, 2013; Sikorski et al., 2023). Of note, all BEIP participants were exposed to this macro-level trend, which does not account for the randomized between‑group differences observed here.

Several factors may limit the interpretation of our findings. First, this study could have benefited from more detailed markers of insulin functioning such as homeostatic model assessment of beta cell (HOMA-β) and insulin function (HOMA-IR), including a suprailia skinfold thickness measure, and the use of magnetic resonance imaging technique to get a more robust measure for body fat composition. Second, the study’s sample size is relatively small, which may have affected the precision of the statistical estimates. In this context, although our four-class BMI trajectory model demonstrated reasonable entropy (0.88), suggesting accurate class separation, the results of the LCGA should be interpreted with caution, as a larger sample size would likely provide higher fit accuracy and greater generalizability (Sinha et al., 2021). Third, while metabolic syndrome may manifest and progress differently by sex, the lack of significant sex differences across trajectory classes suggested limited power to examine sex-specific patterns. Hence, future studies with larger sample sizes are needed to determine whether early deprivation and foster care placement lead to sex-based differences in cardiometabolic health trajectories. Despite these limitations, this unique longitudinal study design enabled us to track the progression of cardiometabolic health from infancy to young adulthood, providing valuable insight into the risk of developing cardiometabolic syndrome in early adulthood.

## Conclusion

Previous BEIP studies have suggested the presence of catch-up growth among those who were randomized into foster care and suggested elevated metabolic risk factors at age 16 (Tang et al., 2018). Leveraging the RCT design of BEIP, this longitudinal study aimed to identify group differences in cardiometabolic traits at age 22 and explore varied risk groups for cardiometabolic syndrome. The study revealed a disadvantage of lipid metabolism among those with a history of institutionalization. Furthermore, individuals in the FCG often have higher BMI and levels of central adiposity compared to those who remained in institution. Individuals with continuous BMI increases from infancy till young adulthood, commonly presented by those in the FCG, displayed poor outcomes across other cardiometabolic traits, such as central adiposity and body fat, and had a higher prevalence of cardiometabolic syndrome. These findings underscore the long-term cardiometabolic consequences of early life adversity and highlight the critical need for early and sustained interventions. Future research is needed to assess whether interventions in childhood and adolescence could improve health outcomes and reduce cardiometabolic risk for individuals with early experience of neglect and foster care.

## Supporting information

10.1017/S0954579426101540.sm001 Mens et al. supplementary material Mens et al. supplementary material

## Data Availability

The data used for this current project can be made available upon request by the PIs of the BEIP (CAN, CHZ, NAF). The program code can be assessed trough https://osf.io/ywzhq/overview?view_only=602f7e46bc08469696929b522e140b24

## Supplementary material

The supplementary material for this article can be found at https://doi.org/10.1017/S0954579426101540.

## Pre-registration statement

This study was not preregistered.
