# Supplementary material for: Long-term cardiometabolic effects of early institutionalization and foster care: Evidence from the Bucharest Early Intervention Project
Source: Dev Psychopathol. 2026 May 15:1–12. Online ahead of print. doi: 10.1017/S0954579426101540 (PMC13222762; doi:10.1017/S0954579426101540)
Supplement: Mens et al. supplementary material [file S0954579426101540sup001.docx]

**
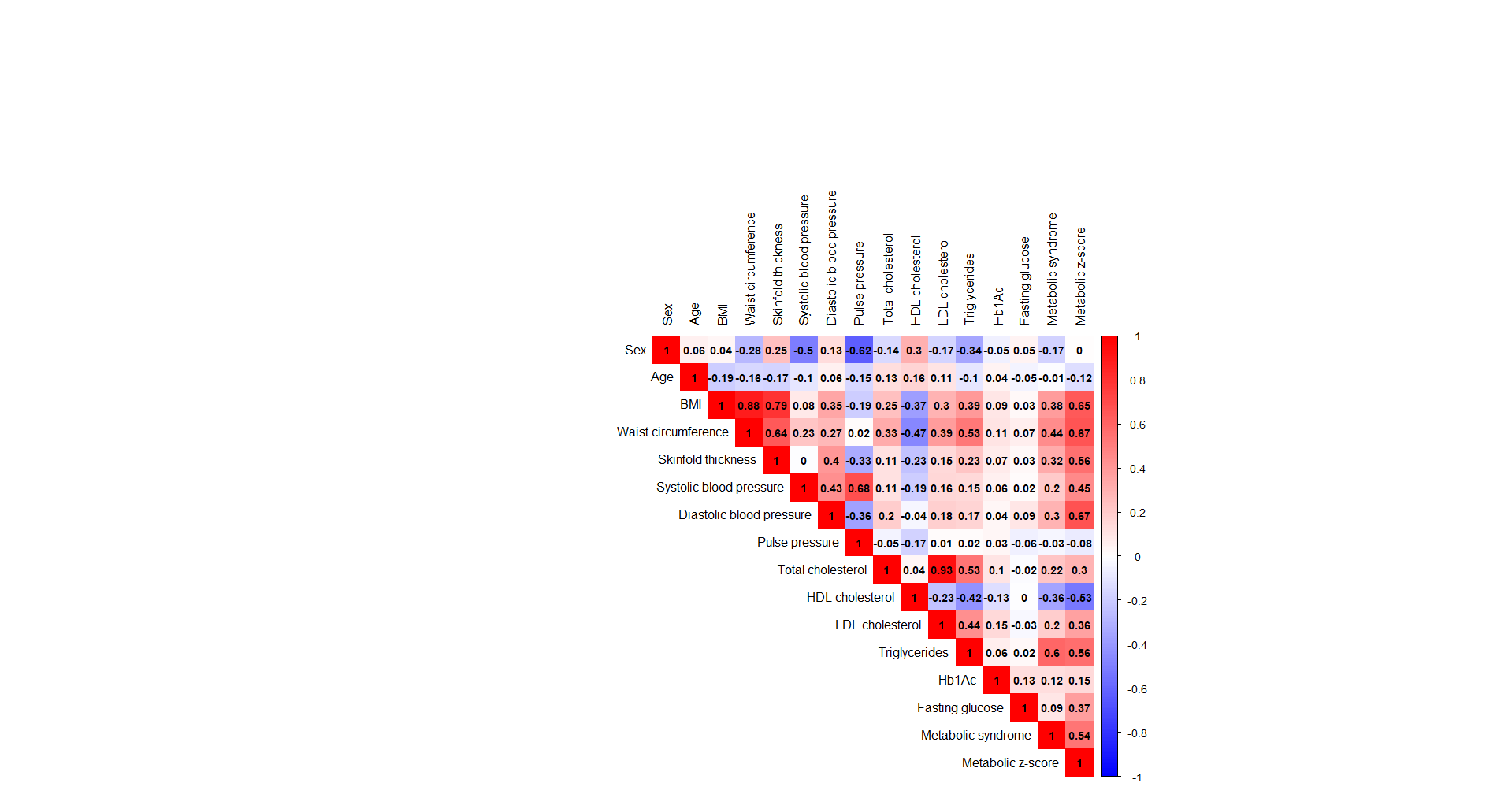
**

**Supplementary Figure 1. Correlation matrix of sex, age and cardiometabolic traits using Pearson correlation**

Measures are from 22-year age assessment


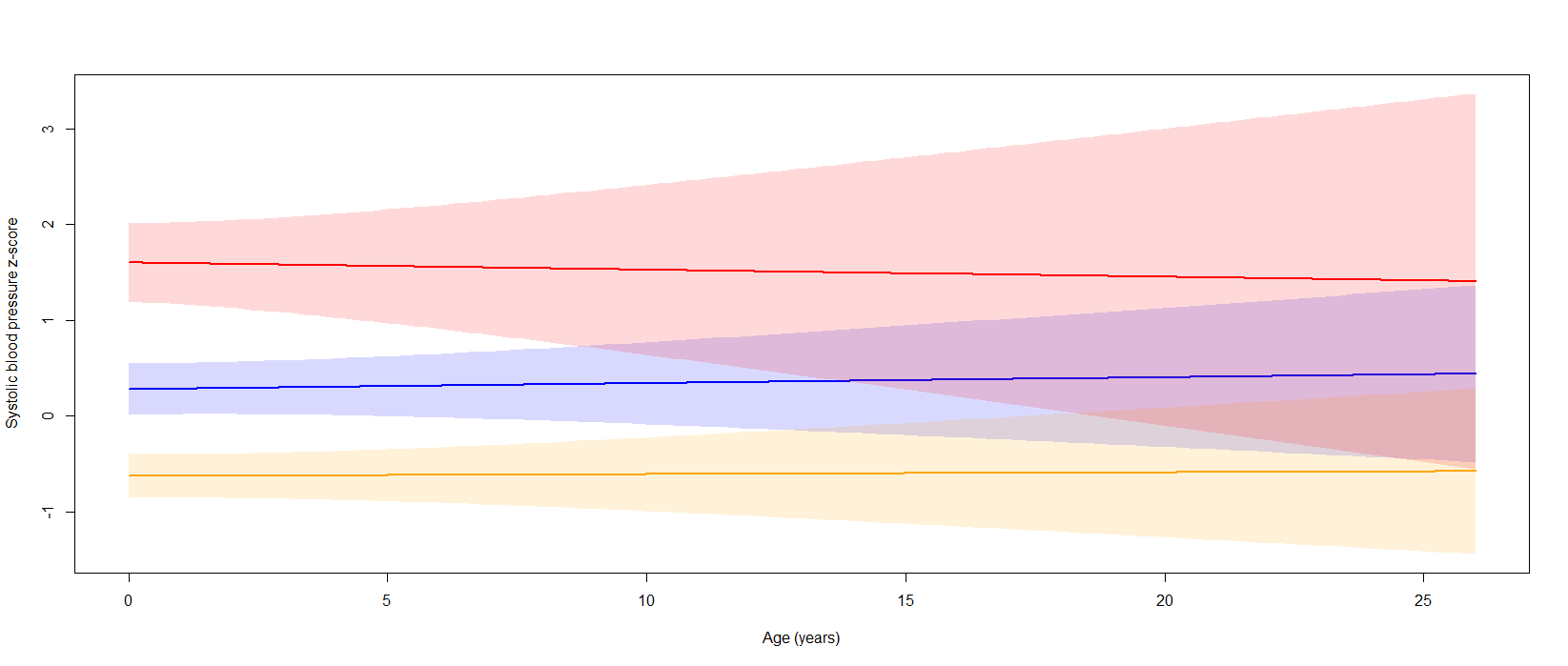


Supplementary Figure 2. Three identified trajectories of the estimated person-mean systolic blood pressure changes from baseline to age 22. Latent growth models identified three heterogeneous trajectories of person-mean centered systolic blood pressure changes over the course of the BEIP study. The largest group is illustrated in blue (n = 57, 48.3%), followed by the group represented in yellow (n = 50, 42.4%), and the group represented in red (n=11, 9.3%).


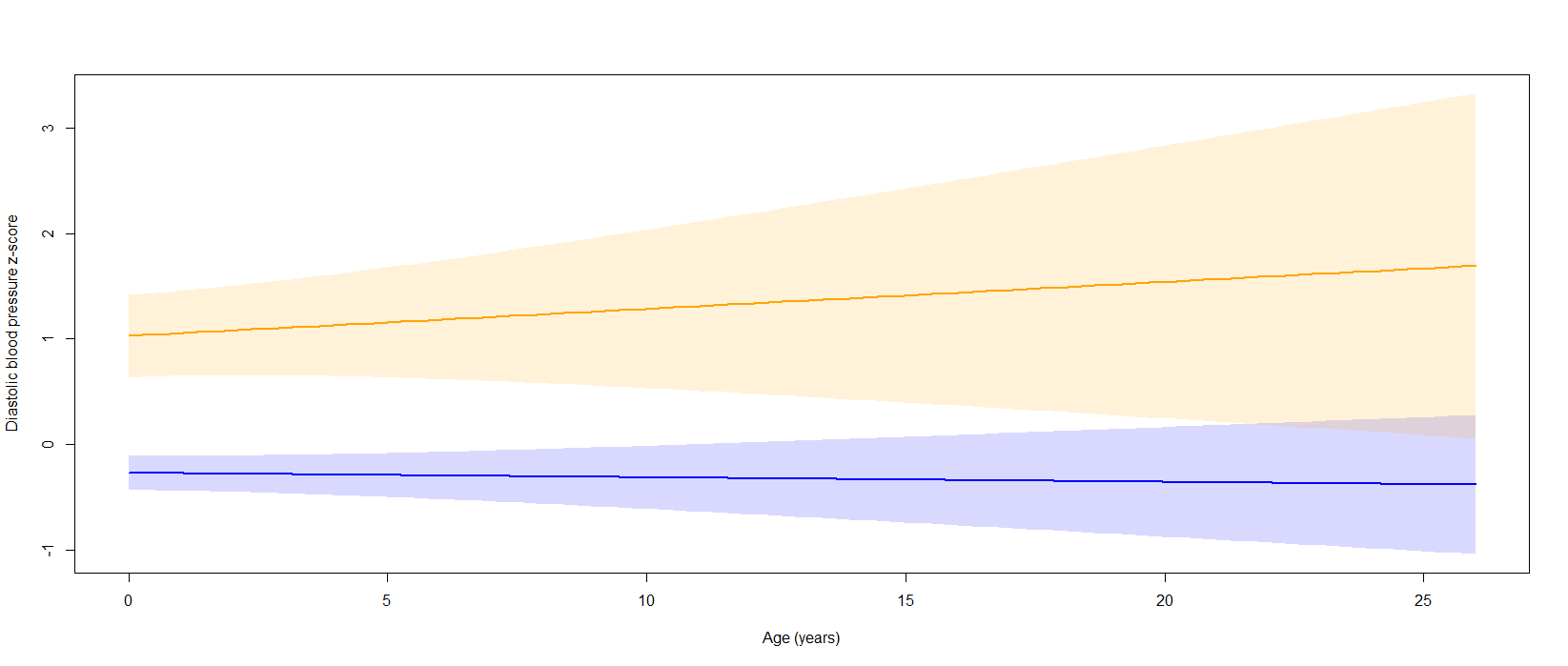


**Supplementary Figure 3.** **Two identified trajectories of the estimated person-mean diastolic blood pressure changes from baseline to age 22.** Latent growth models identified two heterogeneous trajectories of person-mean centered diastolic blood pressure changes over the course of the BEIP study. The largest group is illustrated in blue (n = 97, 82.2%), followed by the group represented in yellow (n = 21, 17.8%).


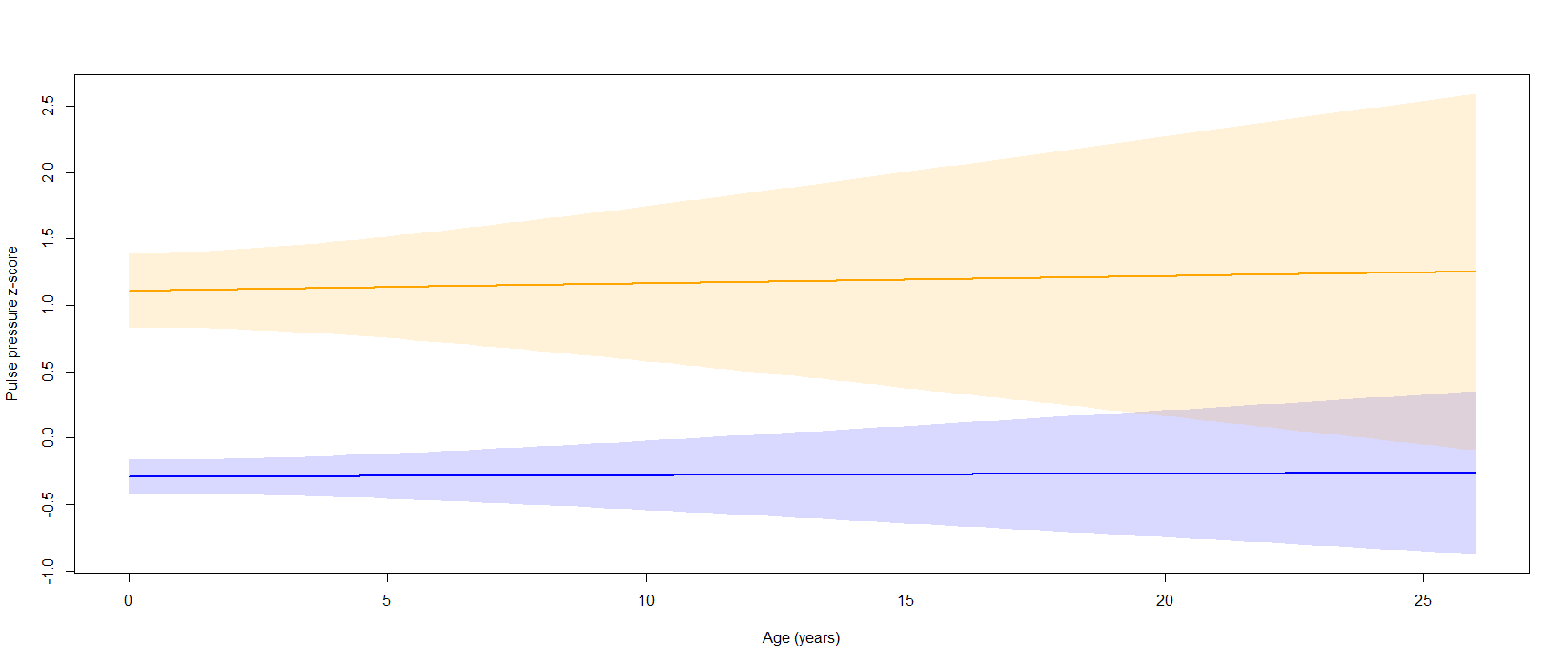


**Supplementary Figure 4.** **Two identified trajectories of the estimated person-mean pulse pressure changes from baseline to age 22.** Latent growth models identified two heterogeneous trajectories of person-mean centered pulse pressure changes over the course of the BEIP study. The largest group is illustrated in blue (n = 92, 78.0%), followed by the group represented in yellow (n = 26, 22.0%).

**Supplementary table 1. Model comparisons for cardiometabolic trait trajectories**

| Trait: Body mass index | |  |  |  |  |  |  |
| --- | --- | --- | --- | --- | --- | --- | --- |
| Model | AIC | BIC | logLik | Deviance | Chi sq | Df | P value Chi sq |
| Linear | 1967.2 | 1986 | -979.62 | 1959.2 |  |  |  |
| Quadratic | 1968.1 | 1991.7 | -979.07 | 1958.1 | 1.0932 | 1 | 0.2958 |
| Cubic | 1969.7 | 1997.9 | -978.84 | 1957.7 | 0.459 | 1 | 0.4981 |
|  |  |  |  |  |  |  |  |
| Trait: Systolic blood pressure | | | | | | | |
| Model | AIC | BIC | logLik | Deviance | Chi sq | Df | P value Chi sq |
| Linear | 848.3 | 863.25 | -420.15 | 840.3 |  |  |  |
| Quadratic | 850.06 | 868.74 | -420.03 | 840.06 | 0.2463 | 1 | 0.6197 |
| Cubic | 851.69 | 874.11 | -419.84 | 839.69 | 0.3705 | 1 | 0.5427 |
|  |  |  |  |  |  |  |  |
| Trait: Diastolic blood pressure | | | | | | | |
| Model | AIC | BIC | logLik | Deviance | Chi sq | Df | P value Chi sq |
| Linear | 854.87 | 869.82 | -423.44 | 846.87 |  |  |  |
| Quadratic | 856.43 | 875.11 | -423.22 | 846.43 | 0.4404 | 1 | 0.50695 |
| Cubic | 854.3 | 876.72 | -421.15 | 842.3 | 4.133 | 1 | 0.04206 |
|  |  |  |  |  |  |  |  |
| Trait: Pulse pressure | | | |  |  |  |  |
| Model | AIC | BIC | logLik | Deviance | Chi sq | Df | P value Chi sq |
| Linear | 856.1 | 871.05 | -424.05 | 848.1 |  |  |  |
| Quadratic | 858.01 | 876.69 | -424 | 848.01 | 0.0959 | 1 | 0.7568 |
| Cubic | 858.47 | 880.89 | -423.24 | 846.47 | 1.5343 | 1 | 0.2155 |

AIC; Akaike Information Criterion, BIC; Bayesian Information Criterion, logLik; Log-Likelihood, Deviance; Model Deviance (Measure of Model Fit), Chi sq; Chi-Square Test Statistic, Df; Degrees of freedom, P value Chi sq; P value for Chi-Square test

**Supplementary table 2. Age at foster care placement and time spent in institution as predictors of cardiometabolic traits**

|  | **Predictor: Age entering foster care (days)** | | | **Predictor: Time spent in institution^*^** | | |
| --- | --- | --- | --- | --- | --- | --- |
| Outcome^†^ | Beta | Std Error | P Value | Beta | Std Error | P Value |
| BMI | 0.00 | 0.01 | 0.79 | 0.09 | 0.08 | 0.26 |
| Waist circumference | 0.00 | 0.01 | 0.89 | 0.19 | 0.19 | 0.34 |
| Skinfold thickness | -0.01 | 0.02 | 0.76 | 0.41 | 0.32 | 0.21 |
| Systolic blood pressure | 0.00 | 0.01 | 0.94 | -0.03 | 0.13 | 0.80 |
| Diastolic blood pressure | -0.01 | 0.01 | 0.46 | -0.09 | 0.13 | 0.50 |
| Pulse pressure | 0.01 | 0.01 | 0.49 | 0.05 | 0.12 | 0.66 |
| Total cholesterol | 0.01 | 0.03 | 0.81 | 0.34 | 0.43 | 0.44 |
| HDL cholesterol | 0.01 | 0.01 | 0.26 | -0.03 | 0.14 | 0.81 |
| LDL cholesterol | 0.00 | 0.02 | 0.88 | 0.35 | 0.39 | 0.37 |
| Triglycerides | -0.04 | 0.03 | 0.28 | 0.09 | 0.55 | 0.88 |
| HbA1c | 0.00 | 0.00 | **0.02** | 0.00 | 0.00 | 0.12 |
| Fasting glucose | 0.00 | 0.01 | 0.62 | -0.20 | 0.13 | 0.13 |
| Metabolic syndrome | 0.00 | 0.00 | 0.76 | 0.00 | 0.00 | 0.43 |
| Metabolic z-score | 0.00 | 0.00 | 0.66 | 0.00 | 0.01 | 0.81 |

Significant p values are highlighted in bold.

* Time spent in an institution as percentage of life till age 18.

^†^ Cardiometabolic outcome measures are from 22-year assessment
Models are adjusted for gender, ethnicity (Roma), smoking, and any medication use.
BMI; body mass index, HDL cholesterol; high-density lipoprotein cholesterol, LDL cholesterol; low-density lipoprotein cholesterol, Hb1Ac; glycosylated hemoglobin.

**Supplementary table 3. Results of linear mixed model; Body mass index**

|  | Ever institutionalized group vs never institutionalized group | | | | Foster care group vs care as usual group | | | | |  |
| --- | --- | --- | --- | --- | --- | --- | --- | --- | --- | --- |
|  |  |  |  |  |  |  |  |  |  |  |
| Body mass index | Model 1 | Model 2 | Model 3 | Model 4 | Model 1 |  | Model 2 | Model 3 | Model 4 |  |
| *Fixed* *effects* |  |  |  |  |  |  |  |  |  |  |
| Intercept | 0.01 (0.07) | 0.01 (0.07) | 0.20 (0.12) | 0.29 (0.25) | -0.07 (0.08) |  | -0.07 (0.08) | -0.26 (0.11) * | -0.07 (0.27) |  |
| Age at assessment |  | -0.00 (0.01) | -0.00 (0.01) | -0.00 (-0.01) |  | | 0.00 (0.01) | 0.00 (0.01) | 0.00 (0.01) |  |
| Institutionalization  (EIG; FCG) | |  | -0.27 (0.15) | **-0.31 (-0.15)*** |  | |  | 0.38 (0.16) * | **0.39 (0.16)*** |  |
| Gender |  |  |  | -0.06 (0.14) |  |  |  |  | -0.16 (0.16) |  |
| Ethnicity (Roma) |  |  |  | 0.10 (0.16) |  |  |  |  | 0.14 (0.17) |  |
| *Random effects* |  |  |  |  |  |  |  |  |  |  |
| Between person variance | 0.47 | 0.30 | 0.30 | 0.30 | 0.50 |  | 0.32 | 0.32 | 0.32 |  |
| Within person variance | 0.52 | 0.55 | 0.53 | 0.53 | 0.54 |  | 0.57 | 0.54 | 0.53 |  |
| *Model* | |  |  |  |  |  |  |  |  |  |
| ICC | 0.53 | 0.69 | 0.69 | 0.69 | 0.52 |  | 0.69 | 0.68 | 0.68 |  |
| N individuals | 132 | 132 | 132 | 132 | 92 |  | 92 | 92 | 92 |  |
| N observation | 813 | 813 | 813 | 813 | 604 |  | 604 | 604 | 604 |  |
|  |  |  |  |  |  |  |  |  |  |  |

EIG; ever institutionalized group, FCG; foster care group
Significant p values are highlighted in bold. P<0.05=*

**Supplementary table 4. Results of linear mixed model; Systolic blood pressure**

| Systolic blood pressure | Ever institutionalized group vs never institutionalized group | | | | Foster care group vs care as usual group | | | |  |
| --- | --- | --- | --- | --- | --- | --- | --- | --- | --- |
|  | Model 1 | Model 2 | Model 3 | Model 4 | Model 1 | Model 2 | Model 3 | Model 4 |  |
| *Fixed effects* |  |  |  |  |  |  |  |  |  |
| Intercept | 0.02 (0.08) | 0.02 (0.08) | -0.06 (0.14) | 0.00 (0.30) | 0.06 (0.78) | 0.06 (0.09) | 0.03 (0.13) | 0.09 (0.32) |  |
| Age at assessment |  | 0.00 (0.01) | 0.00 (0.01) | 0.00 (0.01) |  | 0.00 (0.01) | 0.00 (0.01) | 0.00 (0.01) |  |
| Institutionalization (EIG; FCG) | |  | 0.12 (0.17) | 0.16 (0.18) |  |  | 0.04 (0.18) | 0.04 (0.19) |  |
| Gender |  |  |  | -0.03 (0.15) |  |  |  | -0.02 (0.19) |  |
| Ethnicity (Roma) |  |  |  | -0.16 (0.18) |  |  |  | -0.03 (0.20) |  |
| *Random effects* |  |  |  |  |  |  |  |  |  |
| Between person variance | 0.58 | 0.50 | 0.50 | 0.50 | 0.61 | 0.5 | 0.5 | 0.5 |  |
| Within person variance | 0.44 | 0.47 | 0.47 | 0.47 | 0.48 | 0.52 | 0.52 | 0.52 |  |
| *Model* | |  |  |  |  |  |  |  |  |
| ICC | 0.43 | 0.51 | 0.51 | 0.51 | 0.44 | 0.54 | 0.54 | 0.54 |  |
| N individuals | 118 | 118 | 118 | 118 | 85 | 85 | 85 | 85 |  |
| N observation | 310 | 310 | 310 | 310 | 227 | 227 | 227 | 227 |  |
|  |  |  |  |  |  |  |  |  |  |

EIG; ever institutionalized group, FCG; foster care group

**Supplementary table 5. Results of linear mixed model; Diastolic blood pressure**

| Diastolic blood pressure | Ever institutionalized group vs never institutionalized group | | | | Foster care group vs care as usual group | | | |  | |
| --- | --- | --- | --- | --- | --- | --- | --- | --- | --- | --- |
|  | Model 1 | Model 2 | Model 3 | Model 4 | Model 1 |  | Model 2 | Model 3 | | Model 4 |
| *Fixed effects* | |  |  |  |  |  |  |  | |  |
| Intercept | 0.00(0.07) | 0.00 (0.07) | -0.01(0.13) | 0.01(0.28) | 0.00 (0.09) |  | 0.00 (0.09) | -0.13 (0.13) | | -0.07(0.30) |
| Age at assessment | | 0.00 (0.01) | 0.00 (0.01) | 0.00 (0.01) |  | | -0.00 (0.01) | -0.00 (0.01) | | -0.00 (0.01) |
| Institutionalization (EIG;FCG) | | | 0.01 (0.16) | 0.09 (0.17) |  | |  | 0.26 (0.17) | | 0.23 (0.18) |
| Gender |  |  |  | 0.00 (0.14) |  | |  |  | | 0.00 (0.17) |
| Ethnicity (Roma) | |  |  | -0.26 (0.16) |  | |  |  | | -0.15 (0.18) |
| *Random effects* | |  |  |  |  |  |  |  | |  |
| Between person variance | 0.65 | 0.54 | 0.54 | 0.54 | 0.63 |  | 0.6 | 0.6 | | 0.6 |
| Within person variance | 0.33 | 0.37 | 0.37 | 0.36 | 0.41 |  | 0.42 | 0.41 | | 0.4 |
| *Model* |  |  |  |  |  |  |  |  | |  |
| ICC | 0.34 | 0.45 | 0.45 | 0.44 | 0.39 |  | 0.43 | 0.42 | | 0.42 |
| N individuals | 118 | 118 | 118 | 118 | 85 |  | 85 | 85 | | 85 |
| N observation | 310 | 310 | 310 | 310 | 227 |  | 227 | 227 | | 227 |

EIG; ever institutionalized group, FCG; foster care group

**Supplementary table 6. Results of linear mixed model; Pulse pressure**

| Pulse pressure | Ever institutionalized group vs never institutionalized group | | | | Foster care group vs care as usual group | | | | |
| --- | --- | --- | --- | --- | --- | --- | --- | --- | --- |
|  | Model 1 | Model 2 | Model 3 | Model 4 | Model 1 |  | Model 2 | Model 3 | Model 4 |
| *Fixed effects* | |  |  |  |  |  |  |  |  |
| Intercept | 0.02 (0.07) | 0.02 (0.07) | -0.11 (0.14) | -0.05(0.28) | 0.07 (0.09) |  | 0.07 (0.09) | 0.18 (0.12) | 0.16 (0.30) |
| Age at assessment | | 0.00 (0.01) | 0.00 (0.01) | 0.00 (0.01) |  | | 0.01 (0.01) | 0.01 (0.01) | 0.01 (0.01) |
| Institutionalization (EIG; FCG) | | | 0.17 (0.16) | 0.15(0.17) |  | |  | -0.20 (0.17) | -0.18 (0.18) |
| Gender |  |  |  | -0.04 (0.15) |  | |  |  | -0.02 (0.17) |
| Ethnicity (Roma) | |  |  | 0.07 (0.17) |  | |  |  | 0.14 (0.18) |
| *Random effects* | |  |  |  |  |  |  |  |  |
| Between person variance | 0.64 | 0.56 | 0.56 | 0.56 | 0.67 |  | 0.56 | 0.56 | 0.56 |
| Within person variance | 0.36 | 0.39 | 0.38 | 0.38 | 0.38 |  | 0.42 | 0.42 | 0.41 |
| *Model* |  |  |  |  |  |  |  |  |  |
| ICC | 0.36 | 0.44 | 0.44 | 0.44 | 0.36 |  | 0.47 | 0.47 | 0.46 |
| N individuals | 118 | 118 | 118 | 118 | 85 |  | 85 | 85 | 85 |
| N observation | 310 | 310 | 310 | 310 | 227 |  | 227 | 227 | 227 |

EIG; ever institutionalized group, FCG; foster care group

**Supplementary table 7. Fit indices for class model solutions cardiometabolic trajectories**

| Body mass index | G | loglik | AIC | BIC | SABIC | entropy | %class1 | %class2 | %class3 | %class4 | %class5 |
| --- | --- | --- | --- | --- | --- | --- | --- | --- | --- | --- | --- |
|  | 2 | -1036.84 | 2085.675 | 2102.971 | 2083.993 | 0.90473 | 84.09091 | 15.90909 |  |  |  |
|  | 3 | -988.397 | 1994.793 | 2020.739 | 1992.271 | 0.894258 | 13.63636 | 72.72727 | 13.63636 |  |  |
|  | 4 | -958.731 | 1941.462 | 1976.056 | 1938.1 | 0.876014 | 14.39394 | 11.36364 | 64.39394 | 9.848485 |  |
|  | 5 | -941.941 | 1913.882 | 1957.124 | 1909.679 | 0.877657 | 12.87879 | 15.15152 | 57.57576 | 9.848485 | 4.545455 |
|  |  |  |  |  |  |  |  |  |  |  |  |
| Systolic blood pressure | G | loglik | AIC | BIC | SABIC | entropy | %class1 | %class2 | %class3 | %class4 | %class5 |
|  | 2 | -421.36 | 854.7191 | 871.3432 | 852.3758 | 0.752323 | 82.20339 | 17.79661 |  |  |  |
|  | 3 | -414.984 | 847.9679 | 872.904 | 844.4528 | 0.634696 | 48.30508 | 42.37288 | 9.322034 |  |  |
|  | 4 | -414.621 | 853.2414 | 886.4896 | 848.5547 | 0.547625 | 44.0678 | 15.25424 | 31.35593 | 9.322034 |  |
|  | 5 | -403.873 | 837.7452 | 879.3055 | 831.8868 | 0.645076 | 37.28814 | 13.55932 | 0.847458 | 21.18644 | 27.11864 |
|  |  |  |  |  |  |  |  |  |  |  |  |
| Diastolic blood pressure | G | loglik | AIC | BIC | SABIC | entropy | %class1 | %class2 | %class3 | %class4 | %class5 |
|  | 2 | -422.183 | 856.365 | 872.9891 | 854.0217 | 0.702427 | 82.20339 | 17.79661 |  |  |  |
|  | 3 | -420.274 | 858.5476 | 883.4838 | 855.0326 | 0.497275 | 45.76271 | 46.61017 | 7.627119 |  |  |
|  | 4 | -418.315 | 860.6305 | 893.8787 | 855.9438 | 0.758037 | 79.66102 | 12.71186 | 2.542373 | 5.084746 |  |
|  | 5 | -416.312 | 862.6239 | 904.1842 | 856.7655 | 0.691561 | 0.847458 | 62.71186 | 5.932203 | 24.57627 | 5.932203 |
|  |  |  |  |  |  |  |  |  |  |  |  |
| Pulse pressure | G | loglik | AIC | BIC | SABIC | entropy | %class1 | %class2 | %class3 | %class4 | %class5 |
|  | 2 | -418.497 | 848.9941 | 865.6182 | 846.6508 | 0.766792 | 77.9661 | 22.0339 |  |  |  |
|  | 3 | -416 | 849.9996 | 874.9358 | 846.4846 | 0.777495 | 15.25424 | 77.9661 | 6.779661 |  |  |
|  | 4 | -415.455 | 854.9103 | 888.1585 | 850.2236 | 0.556294 | 22.0339 | 15.25424 | 55.08475 | 7.627119 |  |
|  | 5 | -411.808 | 853.6152 | 895.1754 | 847.7567 | 0.632378 | 31.35593 | 8.474576 | 42.37288 | 15.25424 | 2.542373 |

Akaike Information Criteria (AIC), Bayesian Information Criteria (BIC), and sample size adjusted Bayesian Information Criteria (ssaBIC), integrated complete-data likelihood criterion (ICL).

**Supplementary table 8. Intercept and slopes of identified classes of cardiometabolic trajectories**

| BMI | Class 1 (n=85, 64.4%) | Class 2 (n=19, 14.4%) | Class 3 (n=15, 11.4%) | Class 4 (n=13, 9.8%) | |
| --- | --- | --- | --- | --- | --- |
| Intercept (baseline) | -1.07(0.1)*** | 0.56(0.10)*** | -0.01(0.04)* | 1.58(0.11)*** | |
| Linear slope | 0.03(0.01)** | 0.08(0.01)*** | -0.02(0.00)*** | -0.01(0.01) | |
|  |  |  |  |  |  |
| SBP | Class 1 (n=57, 48.3%) | Class 2 (n=50, 42.4%) | Class 3 (n=11, 9.3%) | | |
| Intercept (baseline) | 0.28(0.14)* | -0.63(0.12)*** | 1.60(0.21)*** | |  |
| Linear slope | 0.006(0.02) | 0.002(0.02) | -0.007(0.04) | |  |
|  |  |  |  |  |  |
| DBP | Class 1 (n=97, 82.2%) | Class 2 (n=21, 18.8%) | | |  |
| Intercept (baseline) | -0.27(0.08)** | 1.03(0.20)*** | |  |  |
| Linear slope | -0.004(0.01) | 0.03(0.03) | |  |  |
|  |  |  |  |  |  |
| PP | Class 1 (n=92, 78.0%) | Class 2 (n=26, 22.0%) | | |  |
| Intercept (baseline) | -0.29(0.06)*** | 1.11(0.14)*** | |  |  |
| Linear slope | 0.001(0.012) | 0.01(0.03) | |  |  |
|  |  |  |  |  |  |
|  |  |  |  |  |  |

Intercept and slopes with (SD). P<0.05=*, p<0.01**, p<0.001***

BMI; body mass index, SBP; systolic blood pressure, DBP; diastolic blood pressure, PP; pulse pressure.

**Supplementary table 9. Characteristics of heterogeneous systolic blood pressure trajectories**

|  | Class 1 | Class 2 | Class 3 | P value |
| --- | --- | --- | --- | --- |
| n | 57 | 50 | 11 |  |
| Age (mean (SD)) | 22.28 (0.92) | 22.78 (1.15) | 22.68 (1.28) | 0.047 |
| Group, n(%) |  |  |  | 0.888 |
| FCG | 22 (38.6) | 17 (34.0) | 5 (45.5) |  |
| CAUG | 18 (31.6) | 19 (38.0) | 4 (36.4) |  |
| NIG | 17 (29.8) | 14 (28.0) | 2 (18.2) |  |
| Ethnicity, n(%) |  |  |  | 0.061 |
| *Romanian* | 43 (75.4) | 33 (66.0) | 7 (63.6) |  |
| *Roma* | 14 (24.6) | 16 (32.0) | 3 (27.3) |  |
| *Hungarian* | 0 (0.0) | 0 (0.0) | 1 (9.1) |  |
| *Other* | 0 (0.0) | 1 (2.0) | 0 (0.0) |  |
| Female, n(%) | 35 (61.4) | 27 (54.0) | 5 (45.5) | 0.541 |
| Smoking, n(%) | 35 (61.4) | 39 (78.0) | 5 (45.5) | 0.054 |
| Medication, n(%) | 3 (5.3) | 0 (0.0) | 1 (9.1) | 0.178 |
| BMI (mean (SD)) | 26.52 (6.06) | 23.95 (5.89) | 25.46 (4.25) | 0.08 |
| Waist circumference (mean (SD)) | 86.71 (13.80) | 82.56 (14.79) | 87.53 (10.99) | 0.26 |
| Skinfold thickness (mean (SD)) | 50.65 (26.03) | 38.90 (24.28) | 39.88 (12.99) | **0.04** |
| Systolic blood pressure (mean (SD)) | 114.18 (9.98) | 103.15 (9.19) | 130.91 (11.25) | **<0.001** |
| Diastolic blood pressure (mean (SD)) | 74.66 (9.25) | 68.35 (7.73) | 81.14 (12.41) | **<0.001** |
| Pulse pressure (mean (SD)) | 39.53 (12.48) | 34.80 (10.12) | 49.77 (10.62) | **<0.001** |
| Total cholesterol (mean (SD)) | 167.71 (32.07) | 159.98 (34.51) | 158.18 (27.09) | 0.408 |
| HDL cholesterol (mean (SD)) | 47.26 (11.33) | 49.36 (13.98) | 49.26 (10.67) | 0.663 |
| LDL cholesterol (mean (SD)) | 100.66 (29.04) | 92.11 (28.85) | 93.58 (24.43) | 0.291 |
| Triglycerides (mean (SD)) | 98.98 (46.63) | 92.55 (61.09) | 76.70 (27.83) | 0.416 |
| Hb1Ac (mean (SD)) | 5.42 (0.37) | 5.39 (0.26) | 5.32 (0.22) | 0.613 |
| Fasting glucose (mean (SD)) | 73.66 (10.55) | 74.02 (8.79) | 75.60 (5.26) | 0.824 |
| Metabolic syndrome, n (%) | 7 (12.3) | 5 (10.0) | 1 (9.1) | 0.911 |
| Metabolic z-score (mean (SD)) | 0.18 (0.53) | -0.25 (0.50) | 0.42 (0.41) | **<0.001** |

Significant p values are highlighted in bold.
FCG; foster care group, CAUG; care as usual group, NIG; never institutionalized group, BMI; body mass index, HDL cholesterol; high-density lipoprotein cholesterol, LDL cholesterol; low-density lipoprotein cholesterol, Hb1Ac; glycosylated hemoglobin.

Cardiometabolic outcome measures are from 22-year assessment

**Supplementary table 10. Characteristics of heterogeneous diastolic blood pressure trajectories**

|  | Class 1 | Class 2 | P value |
| --- | --- | --- | --- |
| n | 97 | 21 |  |
| Age (mean (SD)) | 22.53 (1.08) | 22.55 (1.08) | 0.937 |
| Group, n(%) |  |  | 0.116 |
| FCG | 32 (33.0) | 12 (57.1) |  |
| CAUG | 36 (37.1) | 5 (23.8) |  |
| NIG | 29 (29.9) | 4 (19.0) |  |
| Ethnicity, n(%) |  |  | 0.667 |
| Romanian | 66 (68.0) | 17 (81.0) |  |
| Roma | 29 (29.9) | 4 (19.0) |  |
| Hungarian | 1 (1.0) | 0 (0.0) |  |
| Other | 1 (1.0) | 0 (0.0) |  |
| Female, n(%) | 58 (59.8) | 9 (42.9) | 0.239 |
| Smoking, n(%) | 68 (70.1) | 11 (52.4) | 0.19 |
| Medication, n(%) | 2 (2.1) | 2 (9.5) | 0.295 |
| BMI (mean (SD)) | 24.40 (4.97) | 29.64 (7.96) | **<0.001** |
| Waist circumference (mean (SD)) | 82.58 (11.78) | 96.34 (18.08) | **<0.001** |
| Skinfold thickness (mean (SD)) | 41.92 (21.74) | 57.35 (33.89) | **0.009** |
| Systolic blood pressure (mean (SD)) | 108.73 (11.32) | 121.88 (13.63) | **<0.001** |
| Diastolic blood pressure (mean (SD)) | 70.02 (7.82) | 84.45 (9.31) | **<0.001** |
| Pulse pressure (mean (SD)) | 38.71 (12.00) | 37.43 (12.57) | 0.662 |
| Total cholesterol (mean (SD)) | 161.99 (33.14) | 170.71 (30.43) | 0.27 |
| HDL cholesterol (mean (SD)) | 49.57 (12.79) | 42.66 (8.68) | 0.02 |
| LDL cholesterol (mean (SD)) | 94.82 (28.68) | 103.54 (28.07) | 0.208 |
| Triglycerides (mean (SD)) | 88.03 (49.46) | 122.59 (55.56) | **0.005** |
| Hb1Ac (mean (SD)) | 5.38 (0.32) | 5.48 (0.28) | 0.2 |
| Fasting glucose (mean (SD)) | 73.61 (9.59) | 75.76 (8.41) | 0.344 |
| Metabolic syndrome, n (%) | 6 (6.2) | 7 (33.3) | **0.001** |
| Metabolic z-score (mean (SD)) | -0.12 (0.48) | 0.67 (0.39) | **<0.001** |

Significant p values are highlighted in bold.
FCG; foster care group, CAUG; care as usual group, NIG; never institutionalized group, BMI; body mass index, HDL cholesterol; high-density lipoprotein cholesterol, LDL cholesterol; low-density lipoprotein cholesterol, Hb1Ac; glycosylated hemoglobin.

Cardiometabolic outcome measures are from 22-year assessment

**Supplementary table 11. Characteristics of heterogeneous pulse pressure trajectories**

|  | Class 1 | Class 2 | P value |
| --- | --- | --- | --- |
| n | 92 | 26 |  |
| Age (mean (SD)) | 22.56 (1.12) | 22.42 (0.91) | 0.568 |
| Group, n(%) |  |  | 0.329 |
| FCG | 35 (38.0) | 9 (34.6) |  |
| CAUG | 29 (31.5) | 12 (46.2) | |
| NIG | 28 (30.4) | 5 (19.2) |  |
| Ethnicity, n(%) |  |  | 0.117 |
| *Romanian* | 68 (73.9) | 15 (57.7) | |
| *Roma* | 23 (25.0) | 10 (38.5) | |
| *Hungarian* | 0 (0.0) | 1 (3.8) |  |
| *Other* | 1 (1.1) | 0 (0.0) |  |
| Female, n(%) | 56 (60.9) | 11 (42.3) | 0.144 |
| Smoking, n(%) | 63 (68.5) | 16 (61.5) | 0.669 |
| Medication, n(%) | 4 (4.3) | 0 (0.0) | 0.64 |
| BMI (mean (SD)) | 25.33 (6.42) | 25.35 (3.79) | 0.986 |
| Waist circumference (mean (SD)) | 84.45 (15.18) | 87.09 (8.95) | 0.399 |
| Skinfold thickness (mean (SD)) | 45.52 (26.94) | 41.63 (15.60) | 0.484 |
| Systolic blood pressure (mean (SD)) | 107.67 (11.00) | 123.08 (11.29) | **<0.001** |
| Diastolic blood pressure (mean (SD)) | 72.93 (9.16) | 71.38 (11.86) | 0.48 |
| Pulse pressure (mean (SD)) | 34.74 (9.58) | 51.69 (10.67) | **<0.001** |
| Total cholesterol (mean (SD)) | 164.24 (34.33) | 161.09 (26.71) | 0.666 |
| HDL cholesterol (mean (SD)) | 48.30 (12.45) | 48.48 (12.53) | 0.946 |
| LDL cholesterol (mean (SD)) | 96.70 (29.79) | 95.23 (24.67) | 0.819 |
| Triglycerides (mean (SD)) | 96.24 (54.78) | 86.87 (41.14) | 0.42 |
| Hb1Ac (mean (SD)) | 5.41 (0.34) | 5.33 (0.21) | 0.245 |
| Fasting glucose (mean (SD)) | 74.37 (9.48) | 72.68 (9.13) | 0.422 |
| Metabolic syndrome, n (%) | 10 (10.9) | 3 (11.5) | 1 |
| Metabolic z-score (mean (SD)) | 0.00 (0.58) | 0.10 (0.49) | 0.391 |

Significant p values are highlighted in bold.
FCG; foster care group, CAUG; care as usual group, NIG; never institutionalized group, BMI; body mass index, HDL cholesterol; high-density lipoprotein cholesterol, LDL cholesterol; low-density lipoprotein cholesterol, Hb1Ac; glycosylated hemoglobin.

Cardiometabolic outcome measures are from 22-year assessment
